# New-Onset Henoch–Schonlein Purpura after COVID-19 Infection: A Case Report and Review of the Literature

**DOI:** 10.1155/2022/1712651

**Published:** 2022-03-29

**Authors:** Ashwag Asiri, Faris Alzahrani, Salem Alshehri, Yossef Hassan AbdelQadir

**Affiliations:** ^1^Child Health Department, King Khalid University, Abha City, Saudi Arabia; ^2^Joint Program of Postgraduate Studies in Public Health and Preventive Medicine, Department of Public Health, Ministry of Health, Abha, Saudi Arabia; ^3^Abha Maternity and Children Hospital, Ministry of Health, Abha City, Saudi Arabia; ^4^Alexandria University, Faculty of Medicine, Alexandria, Egypt

## Abstract

Extrapulmonary manifestations of COVID-19 infection include a wide spectrum of cutaneous, endocrine, and cardiovascular complications. We report three cases of new-onset Henoch–Schonlein purpura (HSP) in COVID-19 infected children that were diagnosed and treated in Abha Maternity and Children Hospital, Saudi Arabia, between 28^th^ July 2020 and 10^th^ August 2020. All three cases were males younger than 5 years of age that presented with Henoch–Schonlein purpura characteristic rash and arthralgia without a recent history of any infection, especially respiratory infections. They all tested positive for COVID-19. At the time of the admission, pediatric COVID-19 cases were managed conservatively and we ruled out any other diagnosis before establishing the diagnosis of Henoch–Schonlein purpura according to the clinical picture. The three boys responded significantly to prednisolone and achieved a rapid recovery. We present the clinical scenario and laboratory tests of these children along with pictures of the lesions detected in each case.

## 1. Introduction

Henoch–Schonlein purpura (HSP) is a small blood vessel vasculitis disorder that arises most commonly in the children aged between 3 and 10 years. It is a multiorgan disorder that includes IgA-mediated vasculitis activating the complement system and precipitating an inflammatory response with vasculitis. The incidence of HSP is 10 to 20 cases per 100,000 children per year, and it is diagnosed mainly based on the clinical findings of palpable purpura in addition to one of the following criteria: arthritis or arthralgia, acute onset of abdominal pain, hematuria or proteinuria, and histopathological evidence of leukocytoclastic vasculitis and IgA deposition [1].

Coronavirus disease 2019 (COVID-19) was recognized as a pandemic in early 2020. Since then, several complications were linked to the course of infection including both psychiatric and physical sequalae [2]. COVID-19 is mainly a respiratory infection that ranges in its severity from mild to fatal and it has been associated with multiple extrapulmonary complications and was followed by the new onset of multiple pathologic conditions such as diabetes and acute kidney injury [3]. Recently, individual case reports have reported a possible association between HSP in both children and adults and COVID-19 infection. We report the laboratory and cutaneous findings for three new cases of HSP in COVID-19 infected children that were observed at our care at Abha Maternity and Children Hospital, Abha city, Saudi Arabia between the period of 28^th^ July 2020 and 10^th^ August 2020.

## 2. Case Reports

The protocol of our hospital at that time for each admitted case with fever to be tested for COVID-19 infection: all three cases tested PCR-positive for COVID-19 infection and were isolated according to the protocol. No specific treatment protocol for pediatric COVID-19 cases was developed at this time and all the cases were closely monitored and followed till recovery.

As regards, vaccination status for all the cases was that they did not receive any vaccine before the symptoms because vaccines were not indicated for children at that time in the Kingdom of Saudi Arabia. Also, no skin biopsies were taken from the lesions to identify the characteristic microscopic findings of the vasculitis because the protocol for diagnosis depends on the characteristics clinical findings and exclusion of other causes of bleeding.

Verbal approval was obtained from the parents to publish information about the cases and pictures for the medical literature.

### 2.1. Case 1

**Figure 1:**
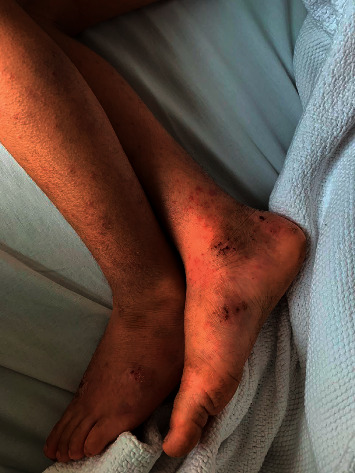
Case 1 shows purpura covering the legs.

A 4-year-old, previously healthy boy was admitted to our hospital on 28^th^ July 2020 after one week of yellow discoloration of eyes and face with dark urine. 4 days before the admission, the mother noticed a small pinpoint rash on the feet without pruritus or insect bites covering both the flexor and extensor surfaces (Figure 1). The next day the child developed a fever spike (38.7 c), bilateral wrist pain and swelling, right knee joint swelling, and inability to walk.

On asking the parents, there was a history of contact with a relative who tested positive for COVID-19. The child had no previous medical or surgical history, except for a history of eczema. He is well vaccinated with no report of allergy to vaccines, foods, or drugs.

On physical examination, the child looked distressed and mildly jaundiced with no signs of anemia or dehydration. There was obvious swelling of the wrist joints, with tenderness and decreased range of motion; the same findings were present in the knee joint, and there was a small purpuric rash on the flexor and extensor surfaces, nonpruritic and nonblanchable.

The child was tested for bilirubin level and serum electrolytes and urine analysis was performed in addition to the routine blood tests. The results showed a CRP level of 0.8 IU/ml and total bilirubin of 2 mg/dl. No abnormalities were detected in serum electrolytes and the patient also tested negative for cytomegalovirus and Epstein–Barr virus.

A diagnosis of HSP was established based on the findings and the child was started on oral prednisolone 2 mg/kg/day, and he immediately improved the next day, the rash on the feet started to fade, the joint pain and swelling decreased, and by the third day, he started to walk. The child was followed for one week and no further complications were reported.

### 2.2. Case 2

**Figure 2:**
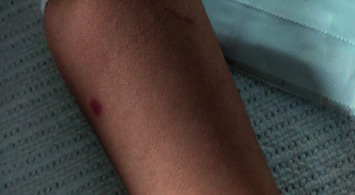
Case 2 shows the skin manifestations detected in the case.

**Figure 3:**
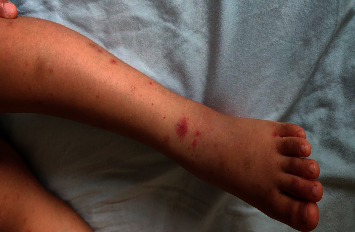
The characteristic leg rash of case 2.

A 23-month-old boy was admitted to our department on 30^th^ July 2020 with a complaint of left-hand swelling and pain and a decrease in the range of motion. This was associated with a purple rash that started to appear on his feet and on the flexor surfaces ascending to reach the buttocks (Figures 2 and 3); he also complained of painful defecation associated with streaks of blood. The patient looked calm on examination with no signs of respiratory distress or dehydration. He was previously admitted 5 days ago with a complaint of bloody stool and severe lower abdominal pain diagnosed as intussusception by abdominal ultrasound. The patient was observed for 2 days, then he was discharged against medical advice.

No history of contact with COVID-19 cases was reported by the parents although the child tested positive by PCR. Routine lab tests showed normal liver function tests, except for a mildly elevated serum alkaline phosphatase. The coagulation profile was normal, CRP was negative, and blood culture was negative for any organism growth.

After excluding bacterial sepsis and bleeding disorders, a diagnosis of HSP was established depending on the clinical picture. The patient received one dose of oral prednisolone from the emergency physician; after that, the pain in his left hand resolved and he no longer experienced bloody stool. Once again, the patient was discharged against medical advice after 1 day.

### 2.3. Case 3

**Figure 4:**
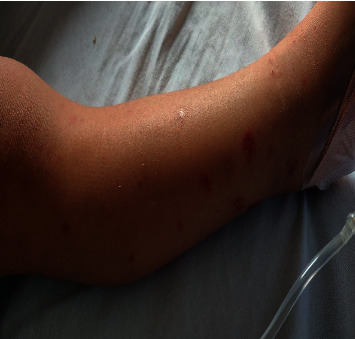
The skin manifestations and joint swelling detected in case 3.

A 4-year-old, previously healthy male was admitted to our hospital on 31st July 2021 with a 9-day history of the wrists, knees, and left ankle joints swelling, pain, and inability to walk. In addition to the joint symptoms, the patient also complained of epigastric intermittent pain, moderate in severity with associated diarrhea 2-3 times per day with blackish stool. At the time of symptoms start, the mother noticed a rash, starting on the feet and ascending to the buttocks, nonpruritic (Figure 4).

No history of contact with COVID-19 cases was reported by the parents although the child tested positive by PCR. Routine lab tests showed a normal coagulation profile and ferritin level. D-Dimer tested positive and stool tested positive for occult blood. All the other lab values including full bleeding profile were normal, except for decreased total protein and albumin levels.

On physical examination, the child was in pain. Yet, he was vitally stable; the knees and left ankle joints were swollen and tender on both active and passive movements.

After excluding hemolysis and infections, a clinical diagnosis of HSP was made and the child was started on prednisolone 2 mg/Kg/day for 5 days; on the next day, the pain disappeared, and the swelling decreased. One day later, he started to eat well and became active.

## 3. Discussion

In this report, we represent 3 cases of new-onset HSP after COVID-19 infection. The three cases were male children, aged less than 4 years. All the cases developed the characteristic palpable, nonblanchable rash of HSP along with the joint symptoms. All three cases responded to prednisolone and were observed till full recovery, except for the second case which was discharged against medical advice after he showed an initial response.

The childhood incidence of COVID-19 is believed to be generally less than adults and carries a better prognosis as well [4]. Several studies have reported cutaneous manifestations associated with the course of COVID-19 infection. These manifestations included purpura, ecchymosis, maculopapular exanthema, and urticarial rash. Results from a study in a tertiary hospital in Pakistan have discovered a significant association between cutaneous manifestations and the severity of the infection. However, these manifestations were generally observed in older patients and they do not carry the specific presentation of HSP present in our study [5].

Vascular endothelial injury and organ vasculitis were detected during COVID-19 infection in several cases. This could have resulted from direct infection of endothelial cells by SARS-CoV-2 or as a result of the inflammatory reaction derived by the infection. Many mechanisms for viral cell invasion are being studied such as the role of angiotensin-converting enzyme 2 (ACE2) receptors and scavenger receptor B type 1 (SR-B1) and other cellular wall receptors that facilitate the entry of the virus to the endothelial cells causing vascular dysfunction and vasculitis [6]. COVID-19 infections were linked to overactive immune responses including the increased levels of inflammatory mediators such as TNF-*α* which in turn induces the production of reactive oxygen species which are known to damage the endothelial cells and cause endothelial dysfunction. It was demonstrated that cytokines level differ according to the stage of the disease and they could represent the basis for the prediction for morbidity duration and mortality in infected patients [6]. Moreover, the evidence suggests that the multiorgan failure reported in the severe cases of COVID-19 infection is caused mainly by the inflammatory response from the body as a result of the invasion of viral particles to the endothelial cells and that is the basis for the recommendations of the guidelines to the use of antiplatelet agents and anticoagulants in severe cases of infection which might reduce the vascular compromise and the risk of microthrombi occluding small vessels in the lungs and other body organs [7].

Upper respiratory tract infection is a known trigger for HSP episodes. The most common organisms are bacterial streptococcal and parainfluenza virus infections, also parvovirus B-19 viral infection. Before the COVID-19 pandemic, coronavirus subtypes were rarely associated with the development of subsequent HSP [8]. However, these are not the first cases to diagnose HSP during or after COVID-19 infection in children. AlGhoozi et al. reported a case of a 4-year-old boy diagnosed with HSP after 37 days of COVID-19 infection. In their report, the child did not require hospital admission for treatment and they excluded other respiratory tract infections during that period which might have triggered the HSP [9]. Another case was documented in a 16-year-old male who presented with hemoptysis and hematochezia in addition to the characteristic lower extremity purpura and abdominal pain [10].

Interestingly, all the observed cases were males who did not develop severe complications associated with HSP. All the cases showed a rapid clinical improvement after treatment. In our reported cases, all the cases received prednisolone and showed a significant improvement within 1 day of administration. As shown in Table 1, all the cases reported showed laboratory values indicating infection. However, there were no serious values in coagulation profile or hemoglobin levels. Urine dipstick analysis from the first case showed mild proteinuria that resolved 1 week after treatment.

Despite the pattern of HSP cases, we reported is supported by previous individual reports as discussed; this article is limited by the lack of COVID-19 vaccination in these cases because all of the reported cases were hospitalized before the vaccine was mandatory for children according to the country's health policy. Also, our results lack the microscopic analysis of a skin lesion biopsy because these were not required for the diagnosis of HSP according to the guidelines of management for such cases in our institution at the time of admission.

Although HSP is usually diagnosed in the pediatric age group, many adult cases of IgA vasculitis were documented in several reports since the onset of the pandemic. In the older age group, the prognosis is usually less favorable than the pediatric age group and some cases develop severe deterioration of renal functions that require much more care [11]. In a case report and literature review of a similar condition in a 70-year-old male by Jedlowski et al., they performed a comprehensive review of 8 adult cases that developed HSP after COVID-19 infection and their results showed that the condition in adults affects males exclusively and that the arthralgia complaint is more severe in adults compared to children [12]. Recently, after vaccinations for COVID-19 are being adapted worldwide, individual case reports have reported a new-onset HSP vasculitis triggered by receiving the first dose of COVID-19 vaccination. According to our literature review, this was observed with 2 types of vaccines: Pfizer‐BioNTech BNT16B2b2 mRNA vaccine and ChAdOx1 nCoV-19 AZD1222 [13, 14].

## 4. Conclusion

This report supports the evidence of the proposed association between COVID-19 infection in children and the increased risk of small vessel vasculitis. We discussed several mechanisms that may have contributed to the development of this complication. Further trials are needed to explore the link between male gender and the development of HSP.

## Figures and Tables

**Table 1:** The results for blood tests, biomarkers, and inflammatory marker levels for the 3 cases.

Lab parameter	Case 1	Case 2	Case 3
Temperature (°C)	36.4	38	36.5
Pulse (beats/min)	99	100	95
Blood pressure (mmHg)	97/53	100/85	100/80
Respiratory rate (per min)	26	30	27
SpO_2_	99%	97%	96%
WBC (x10^3^/uL)	13.18	15.89	8.85
Neutrophils	73%	26.5%	
Lymphocytes	19%	63%	
Monocytes	4.2%	9.4%	
Eosinophils	2.7%	0.7%	
HgB (g/dl)	8.1	11.6	11.8
Platelet count (x10^3^/uL)	476	351	466
ESR (mm)	5	-	12
Lactate dehydrogenase	531		218
INR		1.10	
D-Dimer	-	Positive	Positive
Procalcitonine (ng/ml)	0.11	0.05	0.03

SpO_2_, peripheral blood oxygen saturation; WBC, white blood cell count; HgB, hemoglobin; ESR, erythrocyte sedimentation rate; INR, international normalized ratio.

## Data Availability

No data were used in this study.

## Abbreviations

HSP: Henoch–Schonlein purpura
CRP: C-reactive protein
ACE: Angiotensin-converting enzyme
COVID-19: Coronavirus disease 2019.
